# Extraction-Dependent Antioxidant Activity of Red Horse Chestnut (*Aesculus × carnea*, Family Sapindaceae) Plant Parts

**DOI:** 10.3390/molecules30234550

**Published:** 2025-11-25

**Authors:** Katarzyna Florkowska, Barbara Hanna Roman, Dominika Maciejewska-Markiewicz, Krystyna Cybulska

**Affiliations:** 1 Department of Cosmetic and Pharmaceutical Chemistry, Pomeranian Medical University in Szczecin, Powstańców Wielkopolskich Ave. 72, PL-70111 Szczecin, Poland; katarzyna.florkowska@pum.edu.pl; 2 Department of Human Nutrition and Metabolomics, Pomeranian Medical University in Szczecin, Władysława Broniewskiego Street 24, PL-71460 Szczecin, Poland; dominika.maciejewska@pum.edu.pl; 3 Department of Microbiology, Faculty of Environmental Management and Agriculture, West Pomeranian University of Technology in Szczecin, PL-71434 Szczecin, Poland; krystyna.cybulska@zut.edu.pl

**Keywords:** antioxidant activity, red horse chestnut, medicinal plant, polyphenols, flavonoids

## Abstract

Horse chestnut is a rich source of active compounds that exhibit a variety of biological activities, including antioxidant, anti-inflammatory, and vascular sealing properties. The predominant variety is *Aesculus hippocastanum* L. (White Horse Chestnut), whereas there are limited reports regarding the biological activity, including the antioxidant potential, of the Red Horse Chestnut (*Aesculus carnea* H.) variety. This study aimed to conduct a comprehensive analysis of the antioxidant activity of extracts from various parts of *A. carnea*, including leaves, flowers, unripe, and ripe fruit, as well as to assess the total polyphenol content of the plant, given the limited number of published studies on the subject. This section of the study examined the impact of different extraction factors, including the selection of extraction techniques (Soxhlet extraction, maceration, and ultrasound-assisted extraction) and their parameters (time, solvent type, and concentration). During the second stage of the study, extracts exhibiting the highest antioxidant potential underwent phytochemical analysis utilising HPLC, which included specific phenolic acids and flavonoids. Analyses conducted revealed that extracts from unripe fruits, particularly those prepared in concentrated ethanol using the Soxhlet apparatus, exhibited the highest antioxidant potential and polyphenolic compound content. Notable findings include myricetin (322.281 ± 6.941 mg), 4-hydroxybenzoic acid (25.360 ± 0.525 mg), ferulic acid (62.690 ± 1.350 mg), epicatechin gallate (2.950 ± 0.064 mg), 2-hydroxycinnamic acid (2.013 ± 0.043 mg), ellagic acid (1.735 ± 0.037 mg), and quercetin (1.636 ± 0.037 mg). The antioxidant activity of extracts from unripe fruit, assessed using the DPPH^•^ method, ranged from 0.31 to 3.38 [mg ascorbic acid g^−1^ of fresh raw material]. The results obtained suggest that red horse chestnut, with its significant levels of compounds exhibiting antioxidant potential, such as polyphenols, could serve as a valuable raw material for the pharmaceutical and cosmetics sectors.

## 1. Introduction

The horse chestnut (*Aesculus* L.) is a genus of trees of the subfamily *Hippocastanoideae* of the family *Sapindaceae*. According to taxonomy categorisation, it encompasses 12 to 20 species. Horse chestnuts are indigenous to southern Europe, Asia, and North America [1,2]. In Poland, the common horse chestnut is the most prevalent, although the red horse chestnut *A. carnea* and the yellow horse chestnut *A. flava* are comparatively rare. The primary pharmacological raw material of *Aesculus hippocastanum*, identified by CAS number 8053-39-2, is the seed. However, according to the COSMILE Europe [3] and CosIng [4] databases, the cosmetic raw material encompasses not only the seeds but also the flowers, leaves, bark, and buds. Every component of the horse chestnut (*Aesculus*) plant, encompassing seeds, seedlings, leaves, bark, flowers, and horse chestnut flower honey, is toxic [5]. Nevertheless, horse chestnut is valued in ethnomedicine and as a pharmacological raw material due to the presence of numerous biocompounds [6,7,8,9,10], which exhibit anticancer [11], cardioprotective [12], anti-inflammatory [13,14], neuroprotective [15] and antioxidant effects [14,16]. Particularly important are horse chestnut seeds, which are rich in aescin, which is the main ingredient of preparations used to treat venous diseases [17]. The literature indicates that the horse chestnut (*Aesculus hippocastanum* L.) seeds contain bioactive chemicals that continue to be of interest. However, there are few reports of its undervalued but significant counterpart, the red horse chestnut (*Aesculus carnea*).

**Figure 1:**
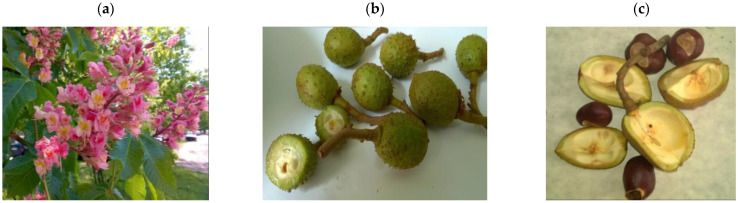
(**a**) Flowers and leaves of the red horse chestnut tree; (**b**) unripe fruits of the red horse chestnut tree; (**c**) ripe fruits and seeds of the red horse chestnut tree.

The European horse chestnut (*Aesculus hippocastanum*) and the American horse chestnut (*Aesculus pavia*) are the two species that have hybridised to produce the red horse chestnut (Figure 1). It is smaller than its European ancestor and has larger grey-brown seeds in a leathery, thornless pericarp that splits into three pieces when ripe, palmately divided compound leaves, and red flowers.

This species has remarkable resilience to environmental degradation, rendering it, alongside its beautiful red flowers, a true ornament of nature. Current data indicates that while the seeds of *Aesculus hippocastanum* and *Aesculus carnea* display morphological and chemical similarities, their pharmacological effects may vary. *A. carnea* seeds possess elevated quantities of aescin, proanthocyanidins and tannins [18] and 20–30% higher concentrations of metals and metalloids in comparison to horse chestnut [19]. Red horse chestnut leaves contain a higher concentrations of polyphenolic compounds than horse chestnut leaves, especially epicatechin and procyanidins [20]. The phytochemical composition of *Aesculus carnea* flower extracts is defined by the presence of flavonoids, flavanols, free phenolic acids, and coumarin [8,21]. The diverse array of active chemicals in red horse chestnut accounts for its multiple effects [8,21,22,23].

There are few reports in the literature regarding the antioxidant properties of *Aesculus carnea*. The purpose of this study was to examine the antioxidant potential of extraction from different parts of *Aesculus carnea* obtained using solvents of varying polarity and extraction methods. The extracts were obtained using a variety of techniques, including the traditional Soxhlet extraction method and the more recent ultrasonic extraction approach, which uses solvents with varying polarity. The antioxidant activity of the extracts was assessed using DPPH^•^, FRAP, and ABTS^•+^ methods, and the total polyphenol concentration was determined using the Folin–Ciocalteu method. Selected extracts were subjected to HPLC analysis in order to identify the components.

## 2. Results and Discussion

The little-known red chestnut is a source of compounds with antioxidant potential, which contributes to its advantageous benefits. Several approaches, including DPPH^•^, ABTS^•+^, and FRAP, facilitate the evaluation of antioxidant activity, signifying that it is a valuable botanical resource.

The antioxidant activity of extracts from leaves, flowers, unripe, and ripe red horse chestnut fruit, assessed using the DPPH^•^ method and expressed as ascorbic acid equivalents [mg of ascorbic acid g^−1^ of fresh raw material], is presented in Table 1. The DPPH^•^ (2,2-diphenyl-1-picrylhydrazyl) method is used to determine the antioxidant capacity of samples through their interaction with the stable DPPH^•^ radical, characterized by an intense purple color. The antioxidant capacity is measured by the decrease in absorbance of the DPPH^•^ solution at a wavelength of 517 nm, indicating the efficacy of the tested compounds in neutralising free radicals through electron or hydrogen atom donation. The DPPH^•^ radical method is commonly employed to assess the antioxidant capacity of phenolic compounds in natural materials such as plant extracts, foods, juices, and fruits. This method offers advantages such as speed, accuracy, and reproducibility. The results are also comparable to those obtained using alternative methods based on free radical scavenging capacity. The results ranged from 0.11 ± 0.05 to 3.41 ± 0.03 mg of ascorbic acid g^−1^ of fresh raw material, corresponding to RSA [%] values ranging from 3.00 ± 1.31 to 93.74 ± 0.76%. In general, in the DPPH^•^ method consistently yielded the highest values for unripe fruit, regardless of the method and solvent. The application of 70% (*v*/*v*) ethyl alcohol in Soxhlet extraction yielded the highest antioxidant activity from unripe fruit extracts. A similar activity was noted in the flower extract using methanol (Soxhlet extraction), as well as in the extract of ripe fruits in acetone, obtained during 60 min ultrasonic extraction. The leaf extract obtained via Soxhlet extraction with acetone demonstrated significant antioxidant activity. The extracts in petroleum ether exhibited the lowest antioxidant potential among all examined parts of the red horse chestnut tree.

The obtained results indicate that the antioxidant activity of extracts from different organs of the red horse chestnut differs statistically significantly. Extracts from unripe fruit demonstrated the highest antioxidant potential, while extracts from ripe fruit and flowers demonstrated lower antioxidant potential. Significant differences between leaf extracts and other parts of the plant confirm that the leaves are also a valuable source of antioxidant compounds.

The FRAP (Ferric Reducing Antioxidant Power) method quantifies the total antioxidant capacity of a sample by assessing its capability to reduce iron(III) ions to iron(II) ions in the presence of the TPTZ complex (2,4,6-tris(2-pyridyl)-s-triazine). The FRAP method quantifies the antioxidant activity of a sample by measuring the colour intensity of the blue Fe^2+^–TPTZ complex spectrophotometrically at 593 nm, which is directly proportional to the concentration of reducing chemicals. This approach is cost-effective, yields reliable results, and the required rea-gents for the reaction are readily synthesized. Another advantage of this method is the rapid analysis. The antioxidant capacity of red horse chestnut extracts, assessed by the FRAP method, ranged from 0.08 ± 0.05 to 77.46 ± 0.10 mg of ascorbic acid g^−1^ of fresh raw material (Table 2). The methanol extract of unripe fruit produced via the Soxhlet method had the highest activity among all samples. The acetone extracts obtained via the Soxhlet method exhibited the greatest values among the leaf extracts, whereas the extracts in petroleum ether displayed the lowest values. A comparable correlation was discovered for extracts from flowers and mature fruit, with the lowest potential identified in samples extracted using petroleum ether. The most effective tech-nique for extracting chemicals with antioxidant properties was a 60 min ultra-sonic extraction for leaves, flowers, and fruit. A comparison of fruits at various ripeness stages indicated that immature fruits had greater antioxidant capacity than ripe fruits, except for acetone extracts derived from the ultrasonic method.

The obtained results show that there are considerable differences in the antioxidant activity of extracts from various red horse chestnut organs. The maximum antioxidant capacity was demonstrated by extracts from unripe fruits, whereas mature fruit and floral extracts demonstrated relatively lower activity. The notable distinctions between leaf extracts and other plant components provide additional evidence that leaves are a substantial source of antioxidant chemicals.

The ABTS^+•^ (2,2′-azino-bis(3-ethylbenzothiazoline-6-sulfonate)) method quantifies the total antioxidant capacity of samples by assessing their capacity to neutralise cationic ABTS^+•^ free radicals. This method evaluates antioxidant activity by measuring the reduction in colour intensity of the ABTS^+•^ solution, using spectrophotometry at approximately 734 nm, which reflects the ability of the test substance to donate electrons or hydrogen atoms. This method offers several advantages, including a high reaction rate of the ABTS^+•^ radical with antioxidants, tolerance across a broad pH range, applicability to both lipophilic and hydrophilic antioxidants, and good solubility of the radical in organic solvents and water. The antioxidant potential of red horse chestnut extracts, evaluated using the ABTS^+•^ method, ranged from 0.03 ± 0.05 to 28.93 ± 0.04 mg of ascorbic acid g^−1^ of fresh raw material (Table 3). The extracts from leaves, flowers, unripe and ripe fruit exhibited the lowest tested properties when extracted with petroleum ether. The extract prepared in 70% (*v*/*v*) ethanol via the Soxhlet apparatus exhibited the highest antioxidant potential among the flower extracts. In the group of ripe fruit extracts, the highest properties were observed for the sample in acetone, 60 min. UAE. Acetone also proved to be the most effective excrement for red horse chestnut leaf extracts using the Soxhlet apparatus. In the case of extracts from unripe fruit, the extract prepared in 70% (*v*/*v*) ethanol using a Soxhlet apparatus exhibited the highest antioxidant potential.

Significant differences in antioxidant potential were observed among extracts from different parts of the red horse chestnut, as measured by the ABTS^•+^ method. Unripe fruits demonstrated the highest antioxidant activity, whereas ripe fruits and flowers exhibited lower activity, confirming organ-dependent variability in antioxidant capacity.

### 2.1. Polyphenol Content in Red Horse Chestnut Extracts Determined by the Folin–Ciocalteu (F-C) Method

The total reducing power of the test sample, which is caused by the presence of phenolic compounds that can reduce the phosphotung-state-phosphomolybdate complex, is measured in order to determine the polyphenol content using the Folin–Ciocalteu reagent method. Sodium carbonate is added to test the presence of phenols at pH 10. According to this method, the amount of polyphenol in the final solution is directly proportional to the intensity of the blue colour, which is determined spectrophotometrically at a wavelength of about 765 nm. This method’s limited specificity is one of its drawbacks, but its simplicity and suitability for standardising biological components are its main advantages.

The total polyphenol content, measured by the F-C method in extracts from leaves, flowers, unripe, and ripe fruits of red horse chestnut was expressed as ascorbic acid equivalents [mg ascorbic acid g^−1^ of fresh raw material] using various extraction methods and solvents and is presented in Table 4. All analysed plant extracts contained polyphenols, with concentrations varying from 0.01 ± 0.01 mg to 7.77 ± 0.07 mg of ascorbic acid g^−1^ of fresh raw material. The methanol extracts obtained through 60 min of ultrasonic extraction from flowers and ripe fruits exhibited the highest concentration of polyphenols. The extracts from unripe fruits exhibited the highest concentration of polyphenols when prepared with concentrated ethanol via a Soxhlet apparatus. In the case of leaf extracts, the highest amount of polyphenols was determined in 70% (*v*/*v*) ethanol using a Soxhlet apparatus. Extracts prepared in petroleum ether were characterized the lowest polyphenol content, suggesting that this solvent is not effective for extracting plant material aimed at obtaining components with high antioxidant properties from the polyphenol group. The optimal time for ultrasound-assisted extraction, considering the total polyphenol content in the extracts from leaves, flowers, ripe and unripe fruit, was determined to be 60 min. The exception were leaf and flower extracts, where a 15 min ultrasonic extraction proved most effective for leaves, and a 30 min extraction for flowers (both extracts were prepared in petroleum ether). Analysis of the total polyphenol content in ripe versus unripe fruit indicates that extracts from unripe fruit typically exhibit higher concentrations. Exceptions included extracts prepared in methanol and acetone using 30- and 60 min ultrasonic extraction, as well as those in petroleum ether using shaking extraction and 60 min ultrasonic extraction.

The leaf extract derived from 70% (*v*/*v*) ethanol by a Soxhlet extractor had the highest total polyphenol concentration among leaves, flowers, unripe fruit, and ripe fruit. Marginally diminished results were obtained for the extract derived from unripe fruit. The Soxhlet extractor was determined to be the most effective technology for digesting this category of antioxidant chemicals in unripe fruit and leaves. However, for ripe fruit and flowers, the highest values were observed in extracts prepared during a 60 min ultrasonic extraction. Similar to other techniques employed to assess antioxidant capability, irrespective of the extraction process and fresh raw material utilised, the lowest values were recorded in extracts obtained with petroleum ether. The results indicate that prolonging the ultrasonic extraction duration to 60 min and employing a Soxhlet extraction device produces extracts with an elevated phenolic component concentration.

Significant differences in total polyphenol content were observed among extracts from different parts of the red horse chestnut. Extracts from unripe fruits contained higher levels of polyphenolic compounds compared to those from ripe fruits and flowers. Additionally, the significant differences observed between leaf extracts and other plant parts confirm that the accumulation of polyphenols in the red horse chestnut is organ-dependent.

### 2.2. Relationships Between the Antioxidant Potential Assessed Using Different Methods for Extracts Prepared from Red Horse Chestnut

Strong and statistically significant correlations between the Folin–Ciocalteu (F-C) method and the antioxidant activity assays (DPPH^•^, FRAP, ABTS^•+^) across all plant parts indicate a close relationship between the total polyphenol content and antioxidant potential (the results are included in the Supplementary Materials, Table S1). The strongest correlations were observed for unripe fruits and flowers, suggesting that phenolic compounds play a dominant role in determining the antioxidant capacity of these plant parts. The subsequent HPLC investigation sought to identify the particular polyphenols that contributed to the reported antioxidant capacity.

### 2.3. HPLC Analysis of Selected Red Horse Chestnut Extracts

The extracts were chosen for their highest antioxidant capabilities. The analysed extracts contained the following polyphenol compounds: 4-hydroxybenzoic acid, ferulic acid, epicatechine gallate, ellagic acid, 2-hydroxycinnamic acid, myrcetin, and quercitin (Table 5). Myrcetin was the chemical found in all extracts, with the highest concentration in E96Sox and the lowest concentration in methanol extracts obtained using ultrasonication and shaking. Ferulic acid and epicatechin gallate were other chemicals identified in the majority of the analysed extracts, with their highest amounts observed in the ethanol extracts obtained with a Soxhlet extractor.

Assessing the effect of the ripeness stage of red horse chestnut fruit on the antioxidant activity of extracts, it can be concluded that extracts from unripe fruit were characterized by the strongest radical scavenging potential, surpassing that of ripe fruit. This conclusion is supported by the findings from both the DPPH^•^ and FRAP methods, irrespective of the extraction method and solvent employed. This high antioxidant potential of extracts derived from unripe fruit is likely associated with the increased presence of antioxidant compounds (flavonoids or polyphenols). Ramalingum and Mahomoodally arrived at comparable conclusions through their analysis of the results obtained, examining the free radical scavenging capacity of extracts derived from the leaves, ripe fruits, unripe fruits, and seeds of the plant *Vangueria madagascariensis* J.F. Gmelin using the DPPH^•^ and FRAP methods [24]. Ntourtoglou et al. conducted an analysis of the polyphenol content in extracts from red horse chestnut leaves using 30 min. ultrasound-assisted extraction with 30% ethanol as a solvent and Pulsed Electric Field (PEF) pretreatment, the amount of which was 62.8 mg g^−1^ GA [20]. The analyzed composition of horse chestnut (*Aesculus hippocastanum*) flower revealed notable antioxidant properties with a capacity of up to 7.85 mmol ascorbic acid equivalent g^−1^). Bioactive compounds, mainly polyphenols, were responsible for these properties, which successfully extracted through fractional methods, achieving a concentration of 414.06 mg g^−1^ [7]. The research conducted by Bielarska et al. [25] involved a comparative analysis of the bioactive compound levels found in the flowers and leaves of horse chestnut (*Aesculus hippocastanum*) and red horse chestnut (*Aesculus × carnea*) over two years (2018 and 2019). Red horse chestnut flowers contained significantly more total carotenoids (40.6 µg g^−1^ dry weight), chlorophylls (36.9 µg/g dry weight), and anthocyanins (5.41 µg g^−1^ dry weight) than horse chestnut flowers. However, the polyphenol content (9.45 µg g^−1^ dry weight) in *A. hippocastanum* flowers was higher than in red horse chestnut flowers. Leaf analysis indicated that red horse chestnut exhibited elevated concentrations of phytochemicals compared to horse chestnut, resulting in greater antioxidant activity than flowers of both species. The findings from our study indicate a consistent pattern of increased antioxidant activity in leaf extracts compared to *A. carnea* flowers, as determined through various methods and solvents; notably, only the application of methanol yielded comparable results. A study by Barreira et al. assessed the antioxidant potential of extracts derived from the flowers, leaves, fruits, and husks of the horse chestnut tree, utilising the DPPH^•^ method for evaluation. The results obtained indicated that the leaves and flowers demonstrated significant antioxidant activity, whereas the fruits displayed even higher values [26]. Gulcin et al. conducted similar observations, extracting the flowers, leaves, fruits, and bark of the horse chestnut tree, followed by an antioxidant activity test utilising the DPPH^•^ radical. The bark extract exhibited the highest antioxidant potential among the tested plant parts, followed by extracts from the fruits, leaves, and flowers [27]. The findings of the previously mentioned authors may corroborate the significant antioxidant capacity of extracts from unripe red horse chestnut fruits identified in our investigation, as well as the variability of antioxidant activity based on the specific plant part utilised. Other research also confirms that antioxidant potential depends on the form of the material (fresh or dried) and the harvest season of the plant material [28,29,30,31].

The selection of extraction parameters might affect the chemical composition of extracts and, consequently, their biological activity. The choice of a suitable solvent results in a variation in the antioxidant potential of extracts derived from red horse chestnut. High antioxidant potential values were reported in methanol, ethanol (96% (*v*/*v*) and 70% (*v*/*v*)), and acetone extracts of ripe and unripe fruits, leaves, and flowers, but the lowest values were recorded for extracts made in petroleum ether. This is due to the solvents’ polarity and its chemical compatibility with the target molecules. Polar solvents like water, ethanol, or methanol are more proficient at extracting hydrophilic chemicals, including phenols and flavonoids. These solvents interact with the molecule via hydrogen bonding. Nonpolar solvents like hexane or chloroform more effectively dissolve lipophilic substances, including essential oils and terpenoids, due to van der Waals and hydrophobic interactions. Medium-polar solvents are also characterised. Acetone exemplifies a solvent regarded as ubiquitous for both polar and nonpolar molecules. Therefore, the optimal extraction solvent may vary depending on the component class and the plant part [32,33].

Different plant organs (e.g., leaves, roots, fruits, seeds) produce and store unique sets and concentrations of bioactive compounds due to their various physiological functions and patterns of gene expression. Furthermore, individual plant parts may produce compounds due to adaptations to their environment, such as those that protect against UV radiation (e.g., pigments) or pathogens (e.g., alkaloids). Additionally, there are differences in the metabolism of compounds in a given tissue (e.g., essential oil synthesis in leaves versus roots). This leads to significant differences in the content of extractable compounds among plant sections, even within the same species [34,35]

The data presented confirm the findings of these studies concerning the effectiveness of the solvents employed (ethanol, methanol, and acetone) in obtaining plant extracts with significant antioxidant properties.

Upon analyzing the effect of the extraction method on the antioxidant activity of red horse chestnut extracts, it can be determined that the most effective technique for isolating antioxidant components is the Soxhlet extraction process and a 60 min ultrasound-assisted extraction. Muzykiewicz et al. examined the efficacy of isolating antioxidant compounds from plant materials utilising a Soxhlet apparatus and a shaking process. They assessed the antioxidant activity of extracts derived from the leaves, ripe, and unripe fruits of rowan (*Sorbus aucuparia* L.) and common quince (*Cydonia oblonga* Mill.), observing that the highest values were achieved for extracts prepared using the Soxhlet apparatus [36]. Bakht et al. propose that ultrasonic extraction is an exceptionally successful technique for obtaining plant extracts rich in flavonoids, and that the antioxidant properties of these extracts are affected by the frequency of the process [37]. The analysis of the extraction method’s impact on the antioxidant capacity of the extracts reveals that red horse chestnut extracts exhibited the highest values when the raw material was in prolonged contact with the solvent and when a new solvent was utilised. We analysed the composition of specific polyphenols utilising HPLC in selected extracts from unripe red horse chestnut fruit. The selection was predicated on the strongest antioxidant activity and a raw material that is somewhat obscure about its active component concentration. Limited research exists about the antioxidant properties of unripe red horse chestnut fruit. Our research indicates that this is a significant raw resource with exceptionally high antioxidant activity, which may potentially serve as a potential medicinal ingredient. The HPLC examination of the chosen extracts identified the presence of the following polyphenols: 4-hydroxybenzoic acid, ferulic acid, epicatechin gallate, ellagic acid, 2-hydroxycinnamic acid, myricetin, and quercetin. Certain chemicals have been identified in other research. Bielarska et al. found ferulic acid, myricetin, and quercetin in the blossoms and leaves of red horse chestnut [25]. Oszmiański et al. also reported the occurrence of polyphenols in flowers and leaves of red horse chestnut [38]. In their study, they identified the presence of quercetin derivatives, epicatechin, in the leaves [38]. Our analysis identified myrcetin as the chemical present in all extracts, with the highest amounts observed in ethanol extracts made using a Soxhlet equipment. These extracts contained markedly higher levels of this compound in comparison to the other samples. A high content myrcetin may be highly advantageous for health perspective [39].

The red horse chestnut is a poorly researched source of bioactive chemicals. It is a source of bioactive chemicals, including polyphenols and flavonoids, which possess significant antioxidant properties. These chemicals are essential as health-promoting agents due to their ability to scavenge free radicals, thereby diminishing oxidative stress [40,41]. Secondary metabolites present in red horse chestnut may inhibit lipid peroxidation, thus preventing vascular diseases and improving the integrity of blood vessels by strengthening capillary walls and reducing their permeability [42]. Therefore, horse chestnut extracts containing, among others, polyphenols contribute to the alleviation of the symptoms of chronic venous insufficiency, acting as an anti-edematous agent and also constricting blood vessels [43,44]. Additionally, the antioxidant activity of this plant also contributes to its anti-aging properties on the skin by scavenging free radicals [42]. Additionally, polyphenols may support wound healing by reducing the risk of bacterial and fungal infections [45] and decreasing inflammation [25].

Among the valuable flavonoids identified in our extracts were quercetin and myricetin. Flavonoids are pigment-like chemicals. The non-sugar part is a 14-carbon skeleton made up of two benzene rings joined by a propane chain. In their glycoside form, they are soluble in water and ethyl alcohol but insoluble in ether, benzene, and chloroform. The pharmacological effects of flavonoids manifest in a variety of ways. They seal and reinforce the capillary walls. As a result, they are utilised as preventative agents for bleeding, varicose veins, petechiae, and atherosclerosis. These effects have been observed in a variety of flavonoid compounds, depending on their chemical structure [46]. These compounds play a crucial role in cellular damage caused by free radicals.

With three rings and five hydroxyl groups, quercitin is a 3,3′,4′,5,7-pentahydroxyflavone with strong antioxidant qualities. A heterocyclic pyrone ring connects quercitin’s two benzene rings, and the molecule’s five hydroxyl groups are responsible for its biological activity [47]. The quercetin effectively neutralizes free radicals, thus protecting the proteins and DNA of damaged cells, particularly those in vascular tissues [41]. Meanwhile, other studies have indicated that quercetin can inhibit key inflammatory pathways such as NF-κB and PI3K/AKT, reducing the production of cytokines and proinflammatory mediators. This compound has been shown to have protective effects in chronic venous insufficiency and acute inflammation [48]. Additionally, quercetin has potential neuroprotective [49] and antidiabetic effects [50].

Myricetin (3,5,7,3′,4′,5′-hexahydroxyflavone) is a significant flavonoid found largely in the glycoside form (O-glycoside). Its antioxidant activity is due to the presence of catechol groups in its structure, which produce semiquinone radicals that can be oxidized [51]. It helps reduce oxidative stress and inflammation, which are linked to chronic diseases such as cardiovascular disease, diabetes, and neurodegeneration [51]. This compound also improves the functioning of blood vessels by reducing platelet aggregation and lowers cholesterol levels, contributing to well heart health [39]. Myricetin helps increase insulin sensitivity, lowers blood glucose levels and reduces lipid levels, so it can be used to support the treatment of diabetes and obesity [52]. Studies confirm the beneficial effect of myricetin on protecting nerve cells from oxidative damage and thus may help prevent neurodegenerative diseases such as Alzheimer’s and Parkinson’s [39]. Myricetin has also been observed to inhibit cancer cell proliferation, induce apoptosis, and inhibit the growth of various human cancer cell lines, including colon, thyroid, and placental cancers, often through modulation of key signalling pathways (e.g., PI3K/Akt/mTOR, MAPK) [53]. Additionally, it also has antimicrobial properties [54].

Specific flavonoids’ chemical characteristics and biological action are determined by their structural diversity. Furthermore, it has been demonstrated that the location and degree of hydroxylation of chromone rings have a substantial impact on the antioxidant capabilities of phenolic compounds. The presence of hydroxyl groups in the ortho position of the benzene ring increases its activity [55].

The multifaceted biochemical action makes the polyphenols, including flavonoids, contained in red horse chestnut valuable for maintaining health, including the cardiovascular system and skin.

The study involved a review and selection of univariate factors. A comprehensive analysis of extracts from various parts of the red horse chestnut tree (leaves, flowers, unripe and ripe fruit) was conducted using solvents of varying polarity (96% and 70% ethyl alcohol, methyl alcohol, acetone, petroleum ether) and various methods.

The evaluation of free radical scavenging ability by DPPH^•^, ABTS^•+^, and FRAP methodologies, together the quantification of polyphenol content, facilitated a comprehensive depiction of antioxidant profiles.

The unripe fruit of the red horse chestnut had the highest antioxidant potential among its extracts. Extracts prepared in 70% (*v*/*v*) ethanol using a Soxhlet apparatus had the greatest antioxidant potential. The HPLC analysis facilitated both qualitative and quantitative assessment of polyphenols present in unripe red horse chestnut fruit. Subsequent research should use extracts from red horse chestnut bark and assess antioxidant potential by the CUPRAC method to achieve a comprehensive understanding of the antioxidant capabilities of this botanical resource.

Preparations obtained from the red horse chestnut tree inhibit lipid peroxidation both enzymatically and non-enzymatically. They also positively impact circulatory system diseases directly related to oxidative stress. Horse chestnut extracts are used internally and externally to treat chronic venous diseases such as edema, varicose veins, and heavy legs, as well as frostbite, burns, and skin inflammation. They exhibit the ability to inhibit prostaglandin synthesis, thereby strengthening and sealing blood vessel walls. Horse chestnut fruit extracts also inhibit the action of lycosomal enzymes, whose activity negatively impacts the condition of veins, causing damage to the mucopolysaccharide layer within capillaries. Fruit extracts also reduce the permeability of electrolytes, water, and proteins through the interstitial tissue. In addition to their vascular protective and anti-edematous effects, they also possess diuretic properties. Given the harmful impact of free radicals on the body, which can lead to autoimmune, neurological, and circulatory system illnesses, it makes sense to avoid and mitigate the damage they do. Under normal settings, free radicals in the cardiovascular system maintain the integrity and function of cardiomyocytes, endothelial cells, and neighbouring smooth muscle cells. However, when the body’s redox balance is disrupted, such as by increased free radical production or diminished antioxidant defence, they play an important role in the development of cardiovascular disease. Cardiovascular disease, in turn, is a major health concern and the leading cause of mortality worldwide. The analysis of the obtained extracts, which have strong antioxidant potential, should be continued and enlarged in the future in order to find and isolate all polyphenolic chemicals that have favourable effects on blood vessel health. These molecules may then be employed as key elements in the development of plant-based medicines, nutraceuticals, and supplements for the prevention and treatment of cardiovascular disease.

## 3. Material and Methods

### 3.1. Plant Material

The plant material used for this study came from the city of Szczecin, Poland (longitude: 14°35′ E, latitude: 53°25′ N). Flowers and leaves of the red horse chestnut were collected in the first half of May, while unripe fruit were collected in June and ripe fruit in September. Fresh plant material: ripe and unripe fruit, leaves, and flowers, was briefly stored in a refrigerator at 4 °C, next was ground and extracted using several methods.

### 3.2. Plant Extracts

The following methods were used to obtain plant extracts: Soxhlet extraction, ultrasound-assisted extraction, and shaking extraction. For all analyses, 5% weight/volume extracts were prepared. The following solvents were used to extract the active ingredients from the plant material: ethyl alcohol 96% (*v*/*v*), ethyl alcohol 70% (*v*/*v*), methyl alcohol 99.8% (*v*/*v*), acetone, and petroleum ether.

#### 3.2.1. Soxhlet Extractor

A measure of 7.50 g of previously prepared plant material (ripe fruit, unripe fruit, leaves, flowers) was weighed. It was then placed in a filter paper thimble and inserted into the Soxhlet extractor (ChemLand, Stargard, Poland). A round-bottomed flask was filled with 150 cm^3^ of the selected solvent (96% (*v*/*v*) ethyl alcohol, 70% (*v*/*v*) ethyl alcohol, 99.8% (*v*/*v*) methyl alcohol, acetone, or petroleum ether) and boiling chips. The plant material was extracted from the moment it began to boil until the selected solvent was poured through the Soxhlet extractor five times. The solution was then filtered through Whatman filter paper (codified EEA03) and placed in tightly sealed dark glass bottles. The extracts were stored at room temperature, protected from light, until further analysis.

#### 3.2.2. Ultrasonic Extraction

A measure of 2.00 g of plant material was placed in a 50 cm^3^ conical flask, 40 cm^3^ of solvent was added, and sonicated in a bath at a frequency of 40 kHz for 15, 30, and 60 min at 40 °C. The extracts were stored in dark bottles at room temperature, protected from light, until further analysis.

#### 3.2.3. Extraction by Shaking

A measure of 2.00 g of plant material was weighed into 100 cm^3^ conical flasks, and 40 cm^3^ of solvent was added. The conical flasks were then placed on a rotary shaker (extraction time: 180 min, rotation frequency: 400 rpm, temperature: 25 °C). The extracts were stored in dark bottles at room temperature, protected from light, until further analysis.

### 3.3. Antioxidant Activity

#### 3.3.1. DPPH^•^ Method

The antioxidant activity of the obtained plant extracts using the DPPH^•^ radical was determined using the method described by Nowak et al. [56] with modifications. DPPH^•^ was prepared by dissolving 12.00 mg of 2,2-diphenyl-1-picrylhydrazyl in 100 cm^3^ of 96% (*v*/*v*) ethanol in a dark glass bottle and stirring for 1 h. The resulting solution was then diluted with 96% (*v*/*v*) ethyl alcohol to obtain an absorbance of 1.00 ± 0.02 at a wavelength of λ = 517 nm. To 2850 μL of the DPPH^•^ solution, 150 μL of the extract or standard solution was added and incubated for 10 min at room temperature. Absorbance measurements were performed at a wavelength of λ = 517 nm. All analyses were performed in triplicate. The obtained values were compared with those for ethanolic ascorbic acid solutions, which were used as a standard to prepare the calibration curve. Antioxidant activity was expressed in mg of ascorbic acid/g of fresh raw material, and the free radical scavenging capacity (RSA [%]) was calculated for each extract or standard sample using the following equation:

RSA[%] = (1 − Ap/Ao) × 100%

where:

Ap—absorbance of the tested extract or standard;

Ao—absorbance of the control sample.

#### 3.3.2. FRAP Method

FRAP analysis of the prepared plant extracts was performed using a modified method proposed by Kucharski et al. [57]. To prepare the working solution, 125 cm^3^ of acetate buffer at pH = 3.6 and a concentration of 0.3 mol dm^−3^, 12.5 cm^3^ of 10 mmol dm^−3^ TPTZ solution in 40 mmol dm^−3^ hydrochloric acid, and 12.5 cm^3^ of iron(III) chloride solution in distilled water were mixed. Next, 80 μL of standard substance or test extract sample was added to 2320 μL of the working solution and incubated for 15 min at room temperature. Sample absorbance was measured at a wavelength of λ = 593 nm in triplicate. The obtained results were compared to the values obtained for ascorbic acid solutions (standard), for which a calibration curve was prepared. All analyses were performed in triplicate.

#### 3.3.3. ABTS^•+^ Method

Determinations using the ABTS**^•+^** reagent were performed using the method described by Kucharski et al. [57], with slight modifications made by the author. A 7 mM solution of 2,2-azino-bis(3-ethylbenzothiazoline-6-sulfonic acid) (ABTS^•+^) was prepared, dissolved in a previously prepared 2.45 mM potassium persulfate solution. The solution was then left in a dark place protected from light for 24 h. After incubation, the ABTS^•+^ solution was diluted with potassium persulfate solution to obtain an absorbance of 0.700 ± 0.005 at a wavelength of λ = 734 nm. To 2499 μL of ABTS^•+^ solution, 25 μL of plant extract or standard solution was added and incubated for 6 min at room temperature. Absorbance measurements were performed at a wavelength of λ = 734 nm. in triplicate. All analyses were performed in triplicate. The obtained values were compared to a calibration curve prepared using ascorbic acid solution as a standard.

#### 3.3.4. Total Polyphenol Content (TPC)

Total polyphenol content in the extracts was determined using a modified method according to Makuch et al. [58]. A measure of 2 mL of Folin–Ciocalteu reagent in 8 mL of water was dissolved in a dark bottle. The solution prepared in this manner was incubated at room temperature for 60 min. To 2700 μL of 5 mmol dm^−3^ Na_2_CO_3_ solution, 150 μL of the extract or standard substance to be tested and 150 μL of the F-C reagent solution were added. The mixture was incubated for 15 min at room temperature. Absorbance measurements were performed at a wavelength of λ = 750 nm. All analyses were performed in triplicate. The results were expressed as ascorbic acid equivalents [mg of ascorbic acid g^−2^ of fresh raw material, and the calibration curve was prepared with ascorbic acid.

#### 3.3.5. HPLC Analysis

The identification of selected polyphenols in the acceptor fluid after permeation, as well as the fluid after skin extraction, was carried out using an HPLC system from Knauer (Berlin, Germany) coupled with the WellChrom UV K-2600 detector (Knauer, Berlin, Germany). The tested components were separated on a 125 × 4 mm column containing Hypersil ODS C18 with a particle size of 5 µm. The column temperature was 25 °C. The phenolic compounds were detected by UV absorption at λ = 278 nm. For the identification of each compound, retention times and comparison with standards under the same conditions were used. The mobile phase consisted of 1% aqueous acetic acid solution (A) and 100% MeOH (B). The samples were eluted with the following gradient: 90% A and 10% B from 0 to 6 min, 84% A and 16% B from 7 to 25 min, 72% A and 28% B from 26 to 37 min, 65% A and 35% B from 38 to 47 min, 50% A and 50% B from 48 to 64 min, and 90% A and 10% B from 65 to 70 min, to recover the initial conditions before injecting a new sample. The flow rate was 0.8 mL min^−1^, and the injection volume was 20 µL. All samples were analyzed three times. The correlation coefficient of the calibration curve was r = 0.998 for 4-hydroxybenzoic acid (y = 16,508x − 1.491, t_R_—10.17 min); r = 0.999 for ferulic acid (y = 2466x − 0.0212, t_R_—33.94 min); r = 0.999 for epicatechine gallate (y =2666x − 0.492, t_R_—34.70 min), r = 0.999 for ellagic acid (y = 15,053x − 0.647, t_R_—41.12 min), r = 0.999 for 2-hydroxycinnamic acid (y = 40,391x − 3.059, t_R_—41.83 min), r = 0.993 for myrcetin (y = 14,908x + 2.145, t_R_—46.76 min), r = 0.998 for quercetin (y = 12,001x + 1.819, t_R_—56.30 min).

### 3.4. Statistical Analysis

The obtained results, taking into account the plant extracts and methods used to assess antioxidant activity, were statistically analyzed. The results are presented as arithmetic means ± standard deviations (SD). For the methods used, results were expressed as ascorbic acid equivalents [mg ascorbic acid/g of fresh raw material]. Additionally, for the DPPH^•^ and ABTS^•+^ methods, RSA values [%] corresponding to ascorbic acid equivalents were calculated. In order to assess the statistical significance between the results obtained by individual methods (DPPH^•^, ABTS^•+^, FRAP and Folin–Ciocalteu methods), Pearson coefficients were determined. The one-way analysis of variance (ANOVA, Tuckey test) grouped the extracts according to the antioxidant assessment method (DPPH^•^, ABTS^•+^, FRAP and Folin–Ciocalteu methods), the type of plant material (leaves, flowers, ripe and unripe fruits), and the solvent (ethyl alcohol 96% (*v*/*v*), ethyl alcohol 70% (*v*/*v*), methyl alcohol 99.8% (*v*/*v*), acetone, and petroleum ether). This allowed us to assess the statistical differences between the results obtained for the different extraction methods. The significance level was α < 0.05. All calculations were done using Statistica 13.1 from StatSoft Polska (StatSoft Polska Sp. z o.o., Kraków, Poland).

## 4. Conclusions

These studies allowed for a comprehensive assessment of the antioxidant activity of red horse chestnut extracts obtained using various methods and solvents. To date, such a comprehensive and exhaustive assessment of the antioxidant potential of red horse chestnut leaves, flowers, and unripe and ripe fruit has not been presented, as there are few literature reports on this topic. Furthermore, unripe fruit was used as the raw material for the study. Various factors influence the antioxidant potential of plant extracts. These include the portion of the plant material used for the study, the solvent used, the extraction method, and the method for determining antioxidant activity. Extracts of red horse chestnut leaves, flowers, ripe and unripe fruit prepared in methanol, 96% (*v*/*v*) and 70% (*v*/*v*) ethanol, and acetone were characterized by very high antioxidant potential. Extracts obtained from unripe horse chestnut fruit demonstrated the highest activity. The type of solvent used significantly influenced the antioxidant activity of the extracts. Polar solvents (ethanol, methanol, acetone) allowed for the production of plant extracts with significantly higher antioxidant potential compared to non-polar solvents (petroleum ether). The extraction conditions used determined the extracts’ properties. The most effective methods were extraction using a Soxhlet extractor and a 60 min ultrasound-assisted extraction. The obtained results highlight the potential use of horse chestnut as a valuable source of natural antioxidants that reduce vascular permeability.

## Figures and Tables

**Table 1:** Mean antioxidant activity (±SD) of extracts from leaves, flowers, unripe and ripe fruits of red horse chestnut, determined using the DPPH^•^ method and expressed as ascorbic acid equivalents [mg of ascorbic acid g^−1^ of fresh raw material], where n = 3. One-way ANOVA was used, followed by Tukey’s post hoc test. Different letters indicate significant differences between individual extracts, α > 0.050.

Extractant	Extraction Methods Used	Ascorbic Acid Equivalent [mg Ascorbic Acid g^−1^ of Fresh Raw Material]			
		Leaves	Flowers	Ripe Fruit	Unripe Fruit
96% (*v*/*v*) ethyl alcohol	Soxhlet extraction	2.87 ± 0.01 ^b^	3.06 ± 0.08 ^a^	2.65 ± 0.01 ^a^	3.36 ± 0.02 ^a^
	Ultrasound 15′	2.46 ± 0.03 ^c^	2.32 ± 0.12 ^c^	0.54 ± 0.01 ^e^	2.00 ± 0.13 ^c^
	Ultrasound 30′	2.79 ± 0.06 ^b^	2.12 ± 0.05 ^d^	1.70 ± 0.05 ^c^	3.10 ± 0.01 ^b^
	Ultrasound 60′	3.00 ± 0.02 ^a^	2.68 ± 0.05 ^b^	1.89 ± 0.02 ^b^	3.32 ± 0.00 ^a^
	Shaking method	2.96 ± 0.02 ^a^	2.52 ± 0.02 ^b^	1.40 ± 0.04 ^d^	3.09 ± 0.01 ^b^
70% (*v*/*v*) ethyl alcohol	Soxhlet extraction	3.22 ± 0.01 ^b^	2.63 ± 0.01 ^c^	3.35 ± 0.00 ^a^	3.41 ± 0.03 ^a^
	Ultrasound 15′	3.28 ± 0.01 ^a^	2.35 ± 0.08 ^d^	1.64 ± 0.02 ^e^	3.35 ± 0.00 ^ab^
	Ultrasound 30′	3.27 ± 0.02 ^ab^	3.02 ± 0.05 ^b^	2.39 ± 0.03 ^d^	3.35 ± 0.02 ^ab^
	Ultrasound 60′	3.22 ± 0.01 ^b^	3.24 ± 0.00 ^a^	2.98 ± 0.02 ^c^	3.32 ± 0.03 ^b^
	Shaking method	2.41 ± 0.03 ^c^	1.29 ± 0.02 ^e^	3.23 ± 0.02 ^b^	3.35 ± 0.02 ^ab^
Methyl alcohol	Soxhlet extraction	3.26 ± 0.01 ^a^	3.40 ± 0.01 ^a^	3.26 ± 0.01 ^ab^	3.38 ± 0.03 ^a^
	Ultrasound 15′	2.76 ± 0.01 ^d^	3.07 ± 0.06 ^c^	1.74 ± 0.01 ^c^	3.31 ± 0.01 ^b^
	Ultrasound 30′	2.93 ± 0.06 ^c^	3.22 ± 0.03 ^b^	3.23 ± 0.01 ^b^	3.33 ± 0.04 ^ab^
	Ultrasound 60′	3.31 ± 0.01 ^a^	3.30 ± 0.01 ^b^	3.25 ± 0.01 ^ab^	3.34 ± 0.00 ^ab^
	Shaking method	3.15 ± 0.02 ^b^	3.27 ± 0.03 ^b^	3.25 ± 0.02 ^a^	3.38 ± 0.02 ^a^
Acetone	Soxhlet extraction	3.32 ± 0.01 ^a^	2.20 ± 0.10 ^a^	2.74 ± 0.02 ^d^	2.90 ± 0.09 ^a^
	Ultrasound 15′	2.68 ± 0.05 ^c^	1.39 ± 0.10 ^c^	2.95 ± 0.05 ^c^	2.47 ± 0.01 ^b^
	Ultrasound 30′	3.08 ± 0.00 ^b^	1.55 ± 0.04 ^bc^	3.05 ± 0.02 ^b^	1.25 ± 0.06 ^c^
	Ultrasound 60′	3.04 ± 0.08 ^b^	1.74 ± 0.11 ^b^	3.37 ± 0.06 ^a^	2.51 ± 0.04 ^b^
	Shaking method	3.01 ± 0.01 ^b^	1.76 ± 0.12 ^b^	2.03 ± 0.03 ^e^	3.04 ± 0.03 ^a^
Petroleum ether	Soxhlet extraction	0.23 ± 0.01 ^c^	0.23 ± 0.00 ^a^	0.18 ± 0.02 ^c^	0.37 ± 0.01 ^b^
	Ultrasound 15′	0.29 ± 0.01 ^b^	0.16 ± 0.03 ^bc^	0.19 ± 0.01 ^c^	0.46 ± 0.03 ^a^
	Ultrasound 30′	0.34 ± 0.01 ^a^	0.19 ± 0.01 ^ab^	0.24 ± 0.01 ^ab^	0.48 ± 0.00 ^a^
	Ultrasound 60′	0.32 ± 0.01 ^ab^	0.11 ± 0.05 ^c^	0.27 ± 0.01 ^a^	0.45 ± 0.03 ^a^
	Shaking method	0.21 ± 0.02 ^c^	0.18 ± 0.01 ^abc^	0.22 ± 0.01 ^b^	0.31 ± 0.01 ^c^

**Table 2:** Average Fe^3+^ ion reduction capacity (±SD) of extracts from leaves, flowers, ripe and unripe fruits of red horse chestnut, determined using the FRAP method and expressed as ascorbic acid equivalents [mg of ascorbic acid g^−1^ of fresh raw material], where n = 3. One-way ANOVA was used, followed by Tukey’s post hoc test. Different letters indicate significant differences between individual extracts, α > 0.050.

Extractant	Extraction Methods Used	Ascorbic Acid Equivalent [mg Ascorbic Acid g^−1^ of Fresh Raw Material]			
		Leaves	Flowers	Ripe Fruit	Unripe Fruit
96% (*v*/*v*) ethyl alcohol	Soxhlet extraction	32.01 ± 0.19 ^a^	17.89 ± 0.04 ^b^	12.73 ± 0.02 ^a^	49.12 ± 0.13 ^a^
	Ultrasound 15′	17.24 ± 0.03 ^e^	14.77 ± 0.03 ^d^	3.83 ± 0.02 ^e^	15.37 ± 0.04 ^b^
	Ultrasound 30’	18.58 ± 0.06 ^d^	14.83 ± 0.06 ^d^	6.96 ± 0.03 ^c^	24.57 ± 0.06 ^b^
	Ultrasound 60’	26.88 ± 0.03 ^c^	18.89 ± 0.03 ^a^	7.42 ± 0.02 ^b^	50.78 ± 17.25 ^a^
	Shaking method	28.92 ± 0.04 ^b^	16.60 ± 0.03 ^c^	5.73 ± 0.02 ^d^	33.73 ± 0.03 ^ab^
70% (*v*/*v*) ethyl alcohol	Soxhlet extraction	64.78 ± 0.10 ^a^	26.24 ± 0.03 ^a^	19.61 ± 0.03 ^a^	65.05 ± 0.11 ^a^
	Ultrasound 15’	29.89 ± 0.04 ^c^	10.24 ± 0.04 ^e^	5.66 ± 0.02 ^e^	27.62 ± 2.48 ^b^
	Ultrasound 30’	42.68 ± 0.14 ^b^	13.25 ± 0.03 ^d^	8.75 ± 0.02 ^d^	36.43 ± 12.60 ^ab^
	Ultrasound 60’	42.89 ± 0.09 ^b^	22.49 ± 0.05 ^b^	11.30 ± 0.02 ^c^	45.79 ± 15.88 ^ab^
	Shaking method	11.04 ± 0.03 ^d^	15.82 ± 0.02 ^c^	16.19 ± 0.04 ^b^	47.46 ± 16.48 ^ab^
Methyl alcohol	Soxhlet extraction	66.67 ± 0.12 ^b^	25.18 ± 0.04 ^a^	12.54 ± 0.02 ^d^	77.46 ± 0.10 ^a^
	Ultrasound 15′	33.82 ± 0.03 ^e^	15.41 ± 0.03 ^e^	7.36 ± 0.01 ^e^	32.28 ± 0.04 ^b^
	Ultrasound 30′	50.85 ± 0.09 ^d^	18.84 ± 0.03 ^d^	35.48 ± 0.14 ^b^	52.40 ± 18.19 ^ab^
	Ultrasound 60′	65.41 ± 0.09 ^c^	22.79 ± 0.04 ^b^	45.46 ± 0.12 ^a^	55.86 ± 19.34 ^ab^
	Shaking method	67.99 ± 0.08 ^a^	22.65 ± 0.03 ^c^	13.22 ± 0.03 ^c^	43.66 ± 15.12 ^ab^
Acetone	Soxhlet extraction	68.22 ± 0.10 ^a^	17.45 ± 0.06 ^a^	13.36 ± 0.03 ^d^	41.85 ± 0.06 ^a^
	Ultrasound 15′	20.95 ± 0.03 ^e^	15.58 ± 0.04 ^c^	23.19 ± 0.02 ^c^	7.58 ± 0.05 ^e^
	Ultrasound 30′	23.30 ± 0.03 ^d^	16.26 ± 0.03 ^b^	50.79 ± 0.14 ^b^	17.32 ± 0.03 ^d^
	Ultrasound 60′	28.59 ± 0.04 ^c^	17.52 ± 0.06 ^a^	57.57 ± 0.10 ^a^	20.12 ± 0.03 ^c^
	Shaking method	42.19 ± 0.08 ^b^	11.59 ± 0.05 ^d^	8.91 ± 0.02 ^e^	32.38 ± 0.03 ^b^
Petroleum ether	Soxhlet extraction	0.69 ± 0.02 ^b^	0.62 ± 0.02 ^ab^	0.11 ± 0.03 ^a^	0.71 ± 0.05 ^a^
	Ultrasound 15′	0.50 ± 0.04 ^b^	0.58 ± 0.02 ^ab^	0.08 ± 0.05 ^a^	0.59 ± 0.02 ^b^
	Ultrasound 30′	0.55 ± 0.03 ^a^	0.63 ± 0.04 ^a^	0.20 ± 0.12 ^a^	0.59 ± 0.04 ^b^
	Ultrasound 60′	0.56 ± 0.04 ^b^	0.66 ± 0.03 ^a^	0.13 ± 0.01 ^a^	0.60 ± 0.02 ^b^
	Shaking method	0.57 ± 0.02 ^b^	0.53 ± 0.05 ^b^	0.14 ± 0.02 ^a^	0.48 ± 0.02 ^c^

**Table 3:** Mean antioxidant activity (±SD) of extracts from leaves, flowers, ripe and unripe fruit of red horse chestnut, determined using the ABTS^+•^ method and expressed as ascorbic acid equivalents [mg of ascorbic acid g^−1^ of fresh raw material], where n = 3. One-way ANOVA was used, followed by Tukey’s post hoc test. Different letters indicate significant differences between individual extracts, α > 0.050.

Extractant	Extraction Methods Used	Ascorbic Acid Equivalent [mg Ascorbic Acid g^−1^ of Fresh Raw Material]			
		Leaves	Flowers	Ripe Fruit	Unripe Fruit
96% (*v*/*v*) ethyl alcohol	Soxhlet extraction	25.89 ± 0.19 ^a^	28.09 ± 0.15 ^a^	16.84 ± 0.17 ^a^	26.33 ± 3.42 ^b^
	Ultrasound 15′	4.59 ± 0.10 ^e^	13.44 ± 0.17 ^d^	2.62 ± 0.15 ^d^	23.49 ± 0.02 ^d^
	Ultrasound 30′	11.44 ± 0.23 ^d^	13.90 ± 0.27 ^cd^	2.90 ± 0.04 ^d^	26.57 ± 0.17 ^c^
	Ultrasound 60′	16.58 ± 0.25 ^c^	17.90 ± 0.19 ^b^	3.34 ± 0.15 ^c^	28.72 ± 0.11 ^a^
	Shaking method	24.76 ± 0.17 ^b^	14.19 ± 0.17 ^c^	4.08 ± 0.13 ^b^	28.71 ± 0.10 ^a^
70% (*v*/*v*) ethyl alcohol	Soxhlet extraction	28.64 ± 0.04 ^a^	28.11 ± 0.10 ^a^	21.44 ± 0.15 ^a^	28.86 ± 0.05 ^a^
	Ultrasound 15′	27.37 ± 0.10 ^c^	10.24 ± 0.19 ^d^	1.30 ± 0.12 ^c^	27.38 ± 0.15 ^c^
	Ultrasound 30′	28.24 ± 0.10 ^b^	13.11 ± 0.24 ^c^	1.57 ± 0.26 ^c^	28.49 ± 0.10 ^b^
	Ultrasound 60′	28.79 ± 0.06 ^a^	20.93 ± 0.18 ^b^	15.72 ± 0.10 ^b^	28.68 ± 0.15 ^ab^
	Shaking method	7.35 ± 0.20 ^d^	5.84 ± 0.17 ^e^	21.08 ± 0.24 ^a^	28.70 ± 0.10 ^ab^
Methyl alcohol	Soxhlet extraction	28.43 ± 0.15 ^a^	25.77 ± 0.16 ^a^	8.26 ± 0.13 ^d^	28.71 ± 0.10 ^a^
	Ultrasound 15′	26.65 ± 0.23 ^c^	12.81 ± 0.17 ^d^	6.24 ± 0.13 ^e^	28.57 ± 0.19 ^a^
	Ultrasound 30′	27.13 ± 0.21 ^b^	13.10 ± 0.17 ^d^	27.78 ± 0.06 ^b^	28.64 ± 0.04 ^a^
	Ultrasound 60′	28.69 ± 0.06 ^a^	19.67 ± 0.31 ^b^	28.64 ± 0.08 ^a^	28.58 ± 0.20 ^a^
	Shaking method	1.23 ± 0.05 ^d^	17.48 ± 0.17 ^c^	13.93 ± 0.19 ^c^	28.79 ± 0.02 ^a^
Acetone	Soxhlet extraction	28.93 ± 0.04 ^a^	17.39 ± 0.25 ^a^	6.23 ± 0.17 ^e^	28.39 ± 0.11 ^a^
	Ultrasound 15′	11.92 ± 0.21 ^d^	3.81 ± 0.18 ^d^	22.84 ± 0.08 ^c^	20.65 ± 0.23 ^c^
	Ultrasound 30′	21.77± 0.24 ^c^	6.23 ± 0.20 ^c^	28.18 ± 0.12 ^b^	23.60 ± 0.23 ^d^
	Ultrasound 60′	22.81 ± 0.17 ^b^	9.41 ± 0.21 ^b^	28.83 ± 0.06 ^a^	24.99 ± 0.11
	Shaking method	28.60 ± 0.08 ^a^	9.10 ± 0.21 ^b^	9.28 ± 0.19 ^d^	23.80 ± 0.09 ^d^
Petroleum ether	Soxhlet extraction	0.08 ± 0.08 ^a^	0.11 ± 0.10 ^a^	0.07 ± 0.06 ^a^	0.04 ± 0.05 ^a^
	Ultrasound 15′	0.17 ± 0.08 ^a^	0.19 ± 0.06 ^a^	0.03 ± 0.05 ^a^	0.04 ± 0.05 ^a^
	Ultrasound 30′	0.12 ± 0.12 ^a^	0.07 ± 0.06 ^a^	0.12 ± 0.12 ^a^	0.04 ± 0.04 ^a^
	Ultrasound 60′	0.10 ± 0.02 ^a^	0.18 ± 0.05 ^a^	0.17 ± 0.11 ^a^	0.06 ± 0.06 ^a^
	Shaking method	0.10 ± 0.09 ^a^	0.28 ± 0.17 ^a^	0.17 ± 0.12 ^a^	0.10 ± 0.10 ^a^

**Table 4:** Mean total polyphenol content (±SD) in extracts from leaves, flowers, ripe and unripe fruits of red horse chestnut, determined using the Folin–Ciocalteu method and expressed as ascorbic acid equivalents [mg ascorbic acid g^−1^ fresh raw material], where n = 3. One-way ANOVA was used, followed by Tukey’s post hoc test. Different letters indicate significant differences between individual extracts, α > 0.050.

Extractant	Extraction Methods Used	Ascorbic Acid Equivalent [mg Ascorbic Acid g^−1^ of Fresh Raw Material]			
		Leaves	Flowers	Ripe Fruit	Unripe Fruit
96% (*v*/*v*) ethyl alcohol	Soxhlet extraction	6.34 ± 0.02 ^b^	5.31 ± 0.02 ^a^	2.27 ± 0.03 ^b^	7.52 ± 0.01 ^a^
	Ultrasound 15′	6.15 ± 0.05 ^c^	4.12 ± 0.05 ^d^	0.84 ± 0.01 ^e^	4.20 ± 0.04 ^e^
	Ultrasound 30′	5.73 ± 0.03 ^e^	3.71 ± 0.01 ^e^	1.78 ± 0.07 ^c^	6.14 ± 0.02 ^d^
	Ultrasound 60′	6.54 ± 0.03 ^a^	4.71 ± 0.05 ^b^	2.48 ± 0.01 ^a^	6.65 ± 0.02 ^b^
	Shaking method	5.99 ± 0.09 ^d^	4.61 ± 0.02 ^c^	1.29 ± 0.03 ^d^	6.26 ± 0.03 ^c^
70% (*v*/*v*) ethyl alcohol	Soxhlet extraction	7.77 ± 0.07 ^a^	5.63 ± 0.09 ^a^	4.48 ± 0.08 ^a^	6.81 ± 0.04 ^a^
	Ultrasound 15′	6.15 ± 0.03 ^c^	2.46 ± 0.04 ^e^	1.85 ± 0.02 ^d^	6.46 ± 0.07 ^b^
	Ultrasound 30′	6.22 ± 0.04 ^c^	3.70 ± 0.05 ^c^	2.77 ± 0.02 ^c^	5.97 ± 0.07 ^c^
	Ultrasound 60′	6.63 ± 0.02 ^b^	5.31 ± 0.02 ^b^	3.60 ± 0.07 ^b^	6.71 ± 0.01 ^a^
	Shaking method	3.31 ± 0.18 ^d^	2.83 ± 0.08 ^d^	4.38 ± 0.03 ^a^	6.68 ± 0.06 ^a^
Methyl alcohol	Soxhlet extraction	6.94 ± 0.02 ^b^	5.59 ± 0.02 ^b^	3.37 ± 0.05 ^b^	7.10 ± 0.02 ^a^
	Ultrasound 15′	5.98 ± 0.07 ^d^	4.42 ± 0.05 ^e^	1.98 ± 0.02 ^d^	6.42 ± 0.07 ^d^
	Ultrasound 30′	5.09 ± 0.07 ^e^	5.09 ± 0.03 ^d^	7.01 ± 0.03 ^a^	6.81 ± 0.04 ^bc^
	Ultrasound 60′	6.75 ± 0.03 ^c^	6.05 ± 0.04 ^a^	7.07 ± 0.02 ^a^	6.89 ± 0.02 ^a^
	Shaking method	7.59 ± 0.10 ^a^	5.28 ± 0.05 ^c^	3.20 ± 0.05 ^c^	6.74 ± 0.04 ^c^
Acetone	Soxhlet extraction	6.94 ± 0.01 ^a^	4.77 ± 0.04 ^a^	2.92 ± 0.02 ^d^	6.36 ± 0.02 ^a^
	Ultrasound 15′	5.02 ± 0.04 ^d^	2.35 ± 0.04 ^d^	4.57 ± 0.06 ^c^	4.76 ± 0.03 ^c^
	Ultrasound 30′	5.32 ± 0.06 ^c^	2.45 ± 0.02 ^d^	5.81 ± 0.03 ^b^	4.65 ± 0.07 ^c^
	Ultrasound 60′	5.41 ± 0.01 ^c^	3.24 ± 0.05 ^c^	6.36 ± 0.04 ^a^	5.07 ± 0.05 ^b^
	Shaking method	6.53 ± 0.08 ^b^	3.46 ± 0.05 ^b^	2.16 ± 0.04 ^e^	6.25 ± 0.04 ^a^
Petroleum ether	Soxhlet extraction	0.01 ± 0.01 ^b^	0.10 ± 0.03 ^a^	0.15 ± 0.02 ^a^	0.35 ± 0.07 ^a^
	Ultrasound 15′	0.09 ± 0.05 ^ab^	0.10 ± 0.02 ^a^	0.12 ± 0.02 ^a^	0.20 ± 0.07 ^bc^
	Ultrasound 30′	0.08 ± 0.04 ^ab^	0.14 ± 0.01 ^a^	0.16 ± 0.00 ^a^	0.15 ± 0.02 ^c^
	Ultrasound 60′	0.05 ± 0.02 ^b^	0.13 ± 0.02 ^a^	0.17 ± 0.02 ^a^	0.29 ± 0.02 ^ab^
	Shaking method	0.16 ± 0.03 ^a^	0.03 ± 0.02 ^b^	0.05 ± 0.01 ^b^	0.03 ± 0.02 ^d^

**Table 5:** The polyphenol content in extracts from unripe red horse chestnut fruit. All values are presented as mean ± SD, where n = 3. One-way ANOVA was used, followed by Tukey’s post hoc test. Different letters indicate significant differences between individual extracts, α > 0.05.

	E96Sox	E96U60	E96U30	E96Sh	E70Sox	E70U60	E70U30	E70Sh	M70Sox	M70U60	M70U30	M70Sh
4-hydroxybenzoic acid	nd	13.765 ± 0.009 ^c^	nd	25.360 ± 0.525 ^f^	nd	8.664 ± 0.115 ^a^	nd	21.720 ± 0.468 ^e^	nd	18.986 ± 0.409 ^d^	nd	12. 055 ± 0.231 ^b^
ferulic acid	nd	nd	nd	6.709 ± 0.071 ^b^	nd	nd	nd	nd	62.690 ± 1.350 ^c^	nd	nd	nd
epicatechine gallate	2.950 ± 0.064 ^e^	0.735 ± 0.01 ^c^	nd	1.299 ± 0.028 ^d^	2.263 ± 0.049 ^e^	0.455 ± 0.010 ^b^	0.841 ± 0.018 ^c^	nd	nd	0.226 ± 0.005 ^a^	2.097 ± 0.045 ^e^	nd
ellagic acid	1.735 ± 0.037 ^g^	0.734 ± 0.016 ^a^	nd	1.039 ± 0.022 ^d^	0.834 ± 0.018 ^b^	0.907 ± 0.020 ^c^	nd	1.110 ± 0.024 ^e^	0.781 ± 0.012 ^a^	1.022 ± 0.022 ^d^	1.228 ± 0.026 ^f^	nd
2-hydroxycinnamic acid	nd	nd	nd	nd	2.013 ± 0.043 ^c^	1.777 ± 0.038 ^b^	nd	nd	nd	nd	nd	0.943 ± 0.020 ^a^
Myrcetin	322.281 ± 6.941 ^f^	14.515 ± 0.528 ^b^	61.485 ± 1.324 ^d^	13.733 ± 0.296 ^b^	40.698 ± 0.876 ^e^	7.515 ± 0.162 ^a^	75.151 ± 1.618 ^d^	12.387 ± 0.267 ^b^	34.05 ± 2.733 ^c^	6.270 ± 0.135 ^a^	4.789 ± 0.103 ^a^	5.701 ± 0.123 ^a^
Quercetin	1.636 ± 0.037 ^c^	nd	nd	nd	0.560 ± 0.012 ^b^	0.400 ± 0.009 ^b^	nd	nd	nd	nd	nd	0.198 ± 0.004 ^a^

nd—no detected, E96Sox—96% (*v*/*v*) ethyl alcohol Soxhlet extraction, E96U60—96% (*v*/*v*) ethyl alcohol ultrasound extraction 60′, E96U30—96% (*v*/*v*) ethyl alcohol ultrasound extraction 30′, E96Sh—96% (*v*/*v*) ethyl alcohol shaking method, E70Sox—70% (*v*/*v*) ethyl alcohol Soxhlet extraction, E70U60—70% (*v*/*v*) ethyl alcohol ultrasound extraction 60′, E70U30—70% (*v*/*v*) ethyl alcohol ultrasound extraction 30′, E70Sh—70% (*v*/*v*) ethyl alcohol shaking method, M70Sox—70% (*v*/*v*) methyl alcohol Soxhlet extraction, M70U60—70% (*v*/*v*) methyl alcohol ultrasound extraction 60′, M70U30—70% (*v*/*v*) methyl alcohol ultrasound extraction 30′, M70Sh—70% (*v*/*v*) methyl alcohol shaking method.

## Data Availability

Data is contained within the article or Supplementary Materials.

## Supplementary Materials

The following supporting information can be downloaded at: https://www.mdpi.com/article/10.3390/molecules30234550/s1, Table S1. A summary of the correlations between the antioxidant assessment methods used for extracts from leaves, flowers, ripe and unripe fruits of A. carnea. Figure S1. Correlations between the antioxidant potential [mg of ascorbic acid g-1 of usable] of red horse chestnut leaf extracts determined using the DPPH, FRAP, ABTS and F-C methods (r—Pearson correlation coefficient, p—application law). Figure S2. Correlations between the antioxidant potential [mg of ascorbic acid g-1 of raw material] of red horse chestnut flower extracts determined using the DPPH, FRAP, ABTS and F-C methods (r—Pearson correlation coefficient, p—probability). Figure S3. Correlations between the antioxidant potential [mg of ascorbic acid g-1 of raw material] of extracts from ripe red horse-chestnut fruits, determined using the DPPH, FRAP, ABTS and F-C methods (r—Pearson correlation coefficient, p—probability. Figure S4. Correlations between the antioxidant potential [mg of ascorbic acid g-1 of raw material] of extracts from unripe red horse-chestnut fruits, determined using the DPPH, FRAP, ABTS and F-C methods (r—Pearson correlation coefficient, p—probability).
