# Supplementary material for: Extraction-Dependent Antioxidant Activity of Red Horse Chestnut (Aesculus × carnea, Family Sapindaceae) Plant Parts
Source: Molecules. 2025 Nov 25;30(23):4550. doi: 10.3390/molecules30234550 (PMC12693346; doi:10.3390/molecules30234550)
Supplement: Supplementary file 1 [file molecules-30-04550-s001.zip › molecules-3943466-supplementary.pdf]

Appendix Data

Extraction-Dependent Antioxidant Activity of Red Horse Chestnut (*Aesculus × carnea*, Family Sapindaceae) Plant Parts.

Katarzyna Florkowska[1], Barbara Hanna Roman[1] , Dominika Maciejewska-Markiewicz[2], Krystyna Cybulska[3]

[1] Department of Cosmetic and Pharmaceutical Chemistry, Pomeranian Medical University in Szczecin, Powstańców Wielkopolskich Ave. 72, PL-70111 Szczecin, Poland; katarzyna.florkowska@pum.edu.pl

[2] Department of Human Nutrition and Metabolomics, Pomeranian Medical University in Szczecin, Władysława Broniewskiego street 24, PL-71460 Szczecin, Poland; dominika.maciejewska@pum.edu.pl

[3] Department of Microbiology and Environmental Chemistry, Faculty of Environmental Management and Agriculture, West Pomeranian University of Technology, Szczecin, PL-71434 Szczecin, Poland; krystyna.cybulska@zut.edu.pl

* Correspondence: barbara.roman@pum.edu.pl

Table S1. A summary of the correlations between the antioxidant assessment methods used for extracts from leaves, flowers, ripe and unripe fruits of *A. carnea*.

	**Leaf extracts**		
	**$ABTS^{\bullet+}$ method**	**FRAP method**	**Folin-Ciocalteu method**
**$DPPH^{\bullet}$ method**	r = 0.807*	r = 0.782*	r = 0.968*
**$ABTS^{\bullet+}$ method**	–	–	r = 0.755*
**FRAP method**	–	–	r = 0.824*
	**Flower extracts**		
	**$ABTS^{\bullet+}$ method**	**FRAP method**	**Folin-Ciocalteu method**
**$DPPH^{\bullet}$ method**	r = 0.869*	r = 0.876*	r = 0.951*
**FRAP method**	–	–	r = 0.943*
	**Extracts from ripe fruits**		
	**$ABTS^{\bullet+}$ method**	**FRAP method**	**Folin-Ciocalteu method**
**$DPPH^{\bullet}$ method**	r = 0.811*	r = 0.704*	r = 0.882*
**$ABTS^{\bullet+}$ method**	–	r = 0.898*	r = 0.932*
**FRAP method**	–	–	r = 0.909*
	**Extracts from unripe fruits**		
		**FRAP method**	**Folin-Ciocalteu method**
**$DPPH^{\bullet}$ method**		r = 0.844*	r = 0.975*
**FRAP method**		–	r = 0.852*

"– " - statically insignificant values, "*" – $p<0.05$

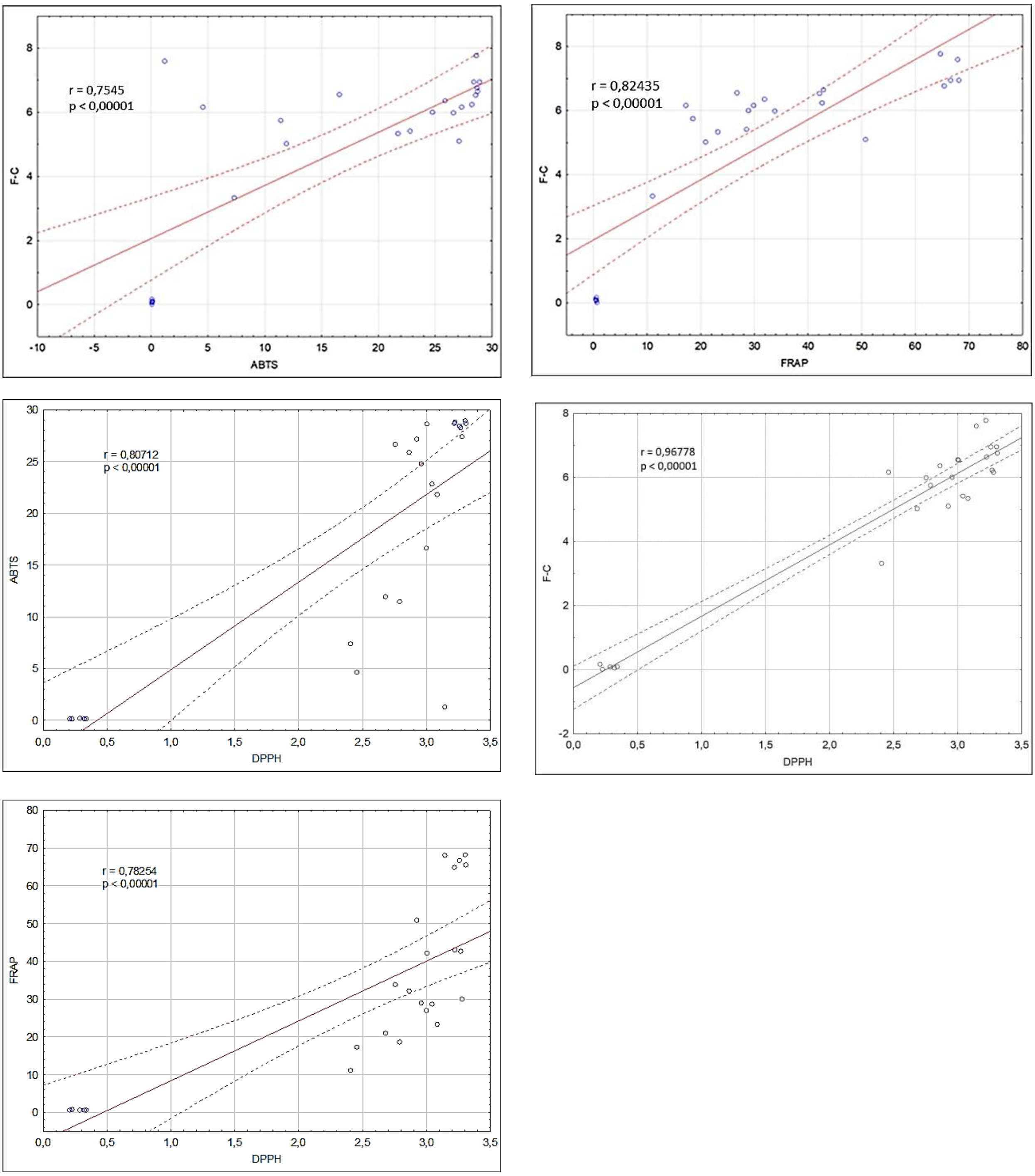

Figure S1. Correlations between the antioxidant potential [mg of ascorbic acid g^{-1} of usable] of red horse chestnut leaf extracts determined using the DPPH, FRAP, ABTS and F-C methods (r – Pearson correlation coefficient, p – application law).

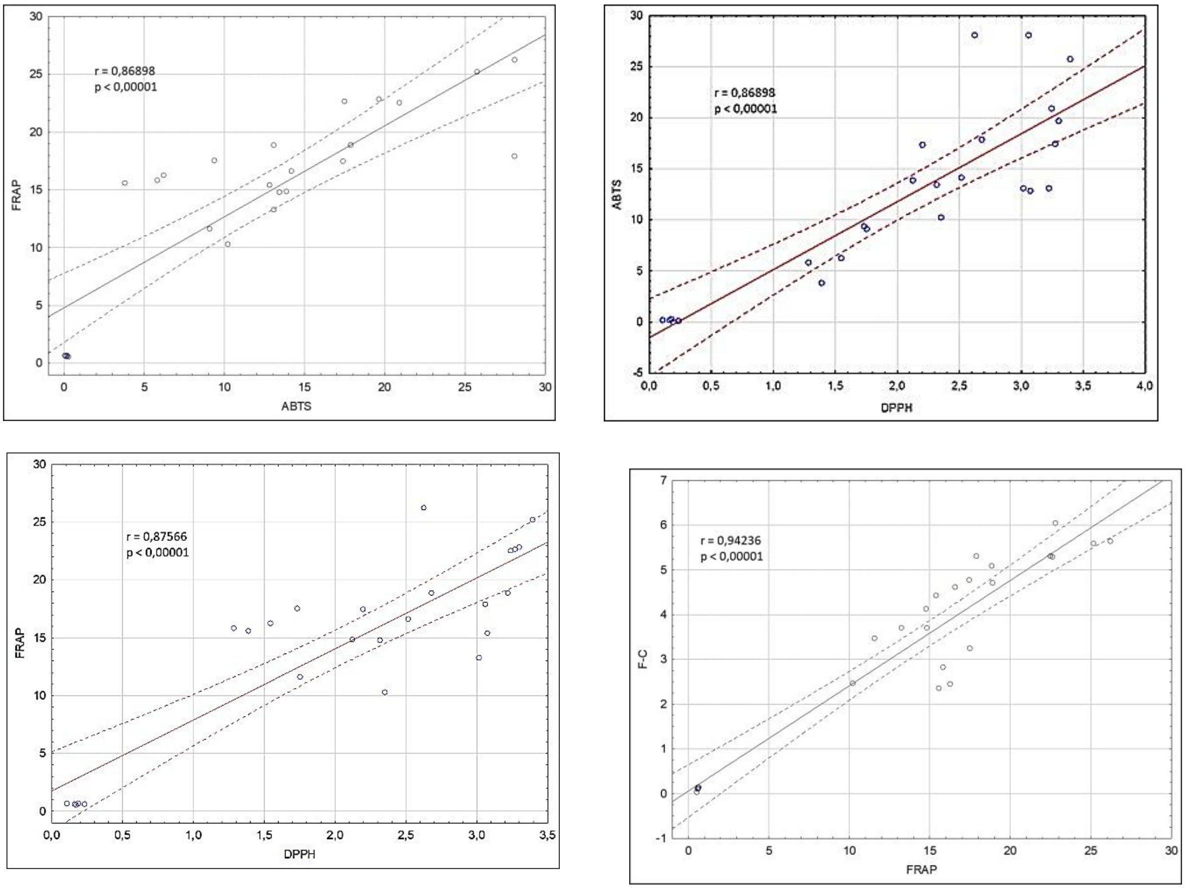

Figure S2. Correlations between the antioxidant potential [mg of ascorbic acid g^{-1} of raw material] of red horse chestnut flower extracts determined using the DPPH, FRAP, ABTS and F-C methods (r – Pearson correlation coefficient, p – probability)

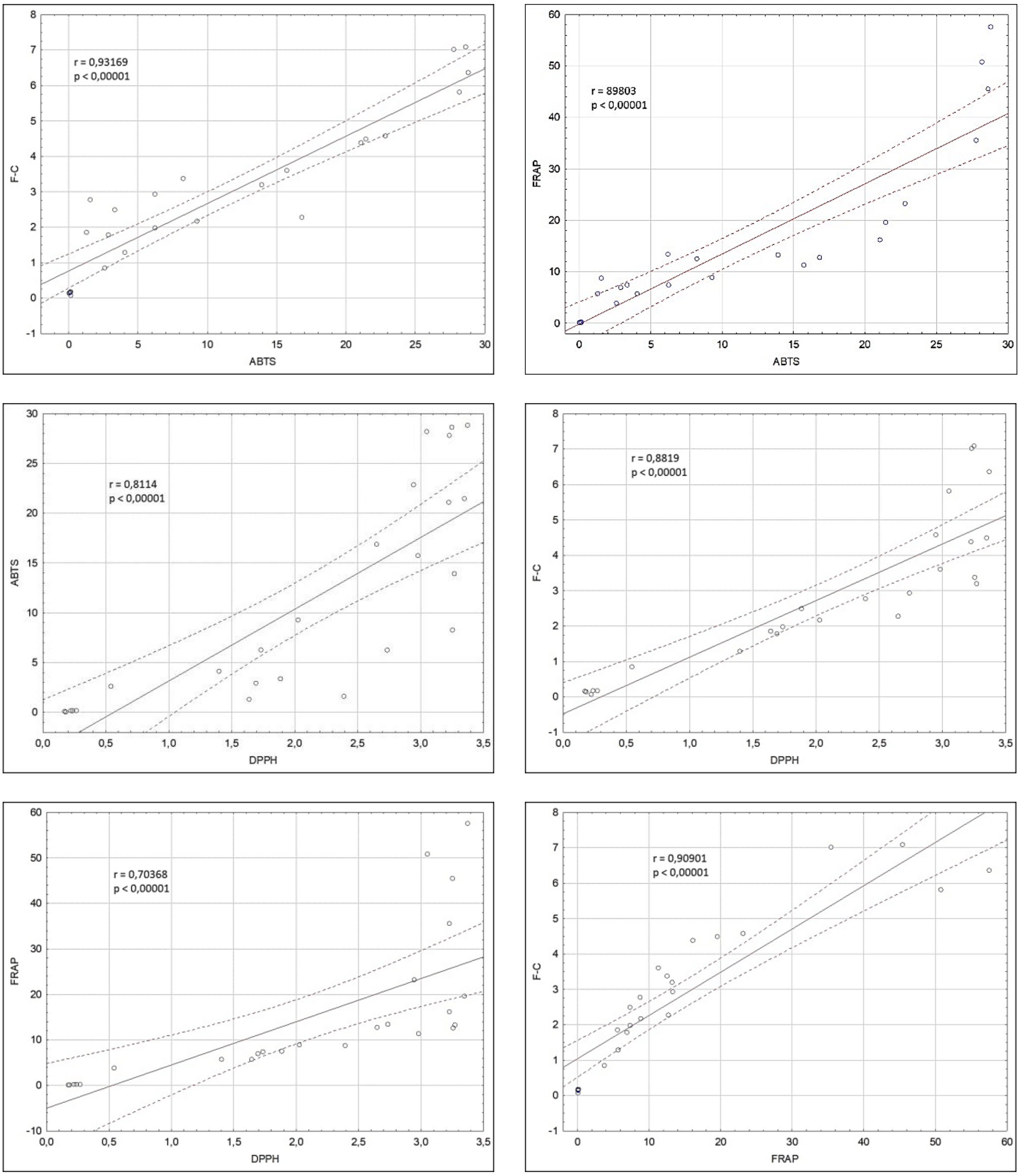

Figure S3. Correlations between the antioxidant potential [mg of ascorbic acid g^{-1} of raw material] of extracts from ripe red horse-chestnut fruits, determined using the DPPH, FRAP, ABTS and F-C methods (r – Pearson correlation coefficient, p – probability

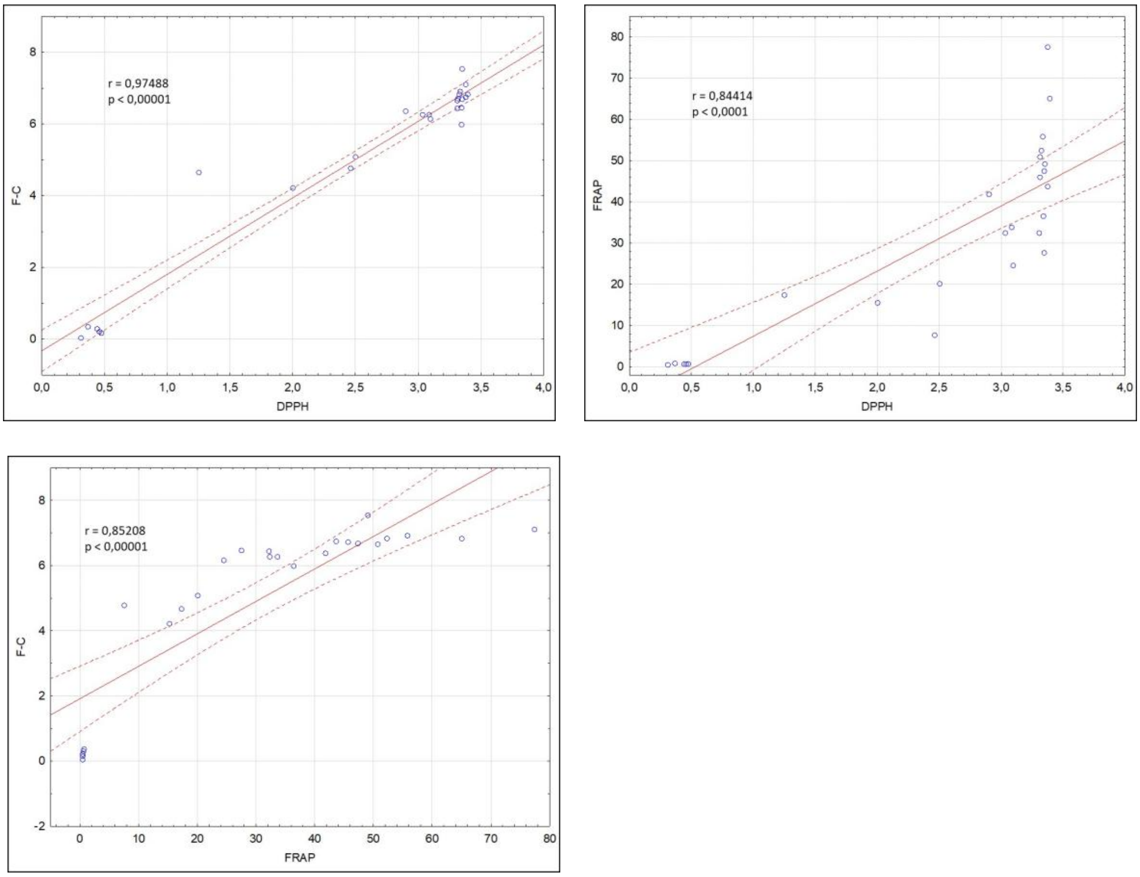

Figure S4. Correlations between the antioxidant potential [mg of ascorbic acid g^{-1} of raw material] of extracts from unripe red horse-chestnut fruits, determined using the DPPH, FRAP, ABTS and F-C methods (r – Pearson correlation coefficient, p – probability).
